# Increasing Coverage of Transcription Factor Position Weight Matrices through Domain-level Homology

**DOI:** 10.1371/journal.pone.0042779

**Published:** 2012-08-27

**Authors:** Brady Bernard, Vesteinn Thorsson, Hector Rovira, Ilya Shmulevich

**Affiliations:** Institute for Systems Biology, Seattle, Washington, United States of America; University of Maryland School of Medicine, United States of America

## Abstract

Transcription factor-DNA interactions, central to cellular regulation and control, are commonly described by position weight matrices (PWMs). These matrices are frequently used to predict transcription factor binding sites in regulatory regions of DNA to complement and guide further experimental investigation. The DNA sequence preferences of transcription factors, encoded in PWMs, are dictated primarily by select residues within the DNA binding domain(s) that interact directly with DNA. Therefore, the DNA binding properties of homologous transcription factors with identical DNA binding domains may be characterized by PWMs derived from different species. Accordingly, we have implemented a fully automated domain-level homology searching method for identical DNA binding sequences.

By applying the domain-level homology search to transcription factors with existing PWMs in the JASPAR and TRANSFAC databases, we were able to significantly increase coverage in terms of the total number of PWMs associated with a given species, assign PWMs to transcription factors that did not previously have any associations, and increase the number of represented species with PWMs over an order of magnitude. Additionally, using protein binding microarray (PBM) data, we have validated the domain-level method by demonstrating that transcription factor pairs with matching DNA binding domains exhibit comparable DNA binding specificity predictions to transcription factor pairs with completely identical sequences.

The increased coverage achieved herein demonstrates the potential for more thorough species-associated investigation of protein-DNA interactions using existing resources. The PWM scanning results highlight the challenging nature of transcription factors that contain multiple DNA binding domains, as well as the impact of motif discovery on the ability to predict DNA binding properties. The method is additionally suitable for identifying domain-level homology mappings to enable utilization of additional information sources in the study of transcription factors. The domain-level homology search method, resulting PWM mappings, web-based user interface, and web API are publicly available at http://dodoma.systemsbiology.netdodoma.systemsbiology.net.

## Introduction

Gene expression is in part regulated by sequence-specific binding of transcription factors (TFs) to target *cis*-regulatory elements in DNA. Therefore, identification of transcription factor binding sites is an essential step in understanding regulatory networks and control in many biological processes, including cellular differentiation, homeostasis, and disease. While experimental studies give a physiologically relevant view of TF-DNA interactions, computational approaches are well suited to enable genome-wide investigation, and to complement and guide further experimental investigation. Accordingly, TF-DNA interactions are commonly described by position weight matrices (PWMs), derived from aligning all known TF binding sequences and log transforming the number of observations of each nucleotide at each position [1], [2]. These provide, through statistical-mechanical theory, a relationship between the observed DNA sequence frequencies used in formulating PWMs and estimates of TF-DNA binding energies [3]. JASPAR [4] and TRANSFAC [5] are two curated databases providing extensive collections of transcription factor PWMs across many species.

The complexity of gene expression patterns is in part due to the combinatorial regulation imparted by multiple TFs acting independently or together under different conditions [6], [7]. While this combinatorial control may arise from direct physical interactions of different domains from multiple TFs, the DNA sequence affinity and specificity of each individual TF, encoded in a PWM, is dictated primarily by select residues within the DNA binding domain(s) of the protein that interact directly with DNA. Therefore, the DNA binding properties of homologous TFs with identical sequences may be characterized by PWMs derived from different species. This is the basis of TfBlast [8], wherein the TRANSFAC database has been connected with BLAST [9] to facilitate TF sequence homology searching. While this is a logical approach to assigning PWMs to identical transcription factors, the homology searching process can be further employed. Since the DNA binding properties of homologous TFs are dictated primarily by their DNA binding domains, homology searching for conserved DNA binding domain sequences may enable cross-species mapping and increased coverage of transcription factor PWMs.

Herein, we describe an approach and pipeline for identifying and mapping PWMs to homologous transcription factors with identical DNA binding domains. These methods have been applied to transcription factors in the JASPAR and TRANSFAC databases in a fully automated manner suitable for genome-scale analyses. We demonstrate the validity of the domain-level homology mapping approach on protein binding microarray data and discuss the resulting increase in coverage in terms of the total number of PWMs associated with each species, as well as the total number of TFs with an assigned PWM, obtained for each PWM database. While the present work focuses on transcription factors with available PWMs, the method is suitable for identifying domain-level homology mappings to enable utilization of additional information sources, such as ChIP data [10] and probabilistic models of TF-DNA interactions [11]. The domain-level homology search method and web API are publicly available at http://dodoma.systemsbiology.netdodoma.systemsbiology.net.

## Methods

### Homology mapping

**Overview:** The DNA binding domains of transcription factors (TFs) were identified and matched to homologous proteins with identical DNA binding domain sequences. The position weight matrices (PWMs) associated with each transcription factor were mapped to all other matches, and the resulting increases in TF-PWM associations were assessed.

**Relating position weight matrices and transcription factor identifiers:** All position weight matrices in JASPAR [4] and TRANSFAC [5] were linked to UniProt Knowledgebase Release 2010_09 identifiers [12]. For JASPAR matrices, the accompanying UniProt identifiers were taken from the http://jaspar.genereg.net/html/DOWNLOAD/all_data/FlatFileDir/matrix_list.txtJASPAR matrix list [http://jaspar.genereg.net/html/DOWNLOAD/all_data/FlatFileDir/matrix_list.txt]. The JASPAR database contains hundreds of motifs for conserved noncoding elements, though these were excluded in the present work as they are not associated in the database with specific transcription factors. For TRANSFAC matrices, the TRANSFAC Factor and Gene identifiers were used to determine the accompanying Swiss-Prot, EMBL, or UniGene identifiers, which were then translated using the ftp://ftp.uniprot.org/pub/databases/uniprot/current_release/knowledgebase/idmapping/UniProt Knowledgebase id mapping [ftp://ftp.uniprot.org/pub/databases/uniprot/current_release/knowledgebase/idmapping/].

**Identifying transcription factor DNA binding domains:** Sequence-specific DNA binding domain identifiers were taken from Vaquerizas et al. [13]. This assembled list primarily consists of DNA binding domains and families from the InterPro database [14] where the authors examined the description and associated literature to assess the sequence-specific DNA binding capabilities. This list was used to identify the DNA binding domain sequence segments, as defined by PROSITE profiles [15], Pfam [16], and SMART [17], [18] databases integrated in InterPro 28.0, for each JASPAR and TRANSFAC matrix-related UniProt identifier. All such sequence segments were selected for TFs with multiple DNA binding domains. In the event that a matrix-related UniProt identifier had no defined DNA binding domains, the entire protein sequence was selected as DNA binding. The selected DNA binding domain sequence segments were then used in the homology search.

**Identifying DNA binding domain matches:** Identical DNA binding domains were identified according to a Position-Specific Iterated BLAST (PSI-BLAST) [9] search of the UniProt Knowledgebase Release 2010_09 [12]. All DNA binding domains from a given query transcription factor were required to have 100% sequence identity, and the DNA binding domain sequence segments were required to have matching lengths, for each DNA binding domain in the query and target transcription factors. The sequence identity and length match threshold has been conservatively set at 100% in the present work. However, this may be reduced in certain cases where the query and target mismatches include residues within the DNA binding domain(s) of the factor that do not affect DNA binding. Predetermined mappings and user defined queries with more permissive sequence identity and length match thresholds are available through the web server or API at http://dodoma.systemsbiology.netdodoma.systemsbiology.net.

The accompanying JASPAR and TRANSFAC matrix identifiers for each query and target transcription factor match were recursively associated. For example, consider the case where TFs A and B are associated with PWMs 1 and 2, respectively. Through the domain matching process, TFs A and B are found to have matching DNA binding domains. Now TFs A and B are each recursively associated with PWMs 1 and 2. Subsequently in the homology search, transcription factor C is also found to have a matching DNA binding domain, resulting in the recursive association of C with PWMs 1 and 2.

As a point of comparison for increased PWM coverage resulting from homology, the present domain-level search was compared to 100% sequence identity and length matches and recursive PWM associations for the complete TF sequence.

**Accounting for redundancy:** Within the UniProt Knowledgebase, there are cases where multiple identifiers are associated with the same transcription factor or splice variants of a given transcription factor within a species. In these cases, the complete cross-referencing between UniProt, JASPAR, and TRANSFAC identifiers has been conducted. However, multiple query-target matches from the same species were not counted as new unique matches for analysis purposes; only cross-species DNA binding domain homology matches were considered.

### Validation of domain-level TF-DNA specificities

**Figure 1:**
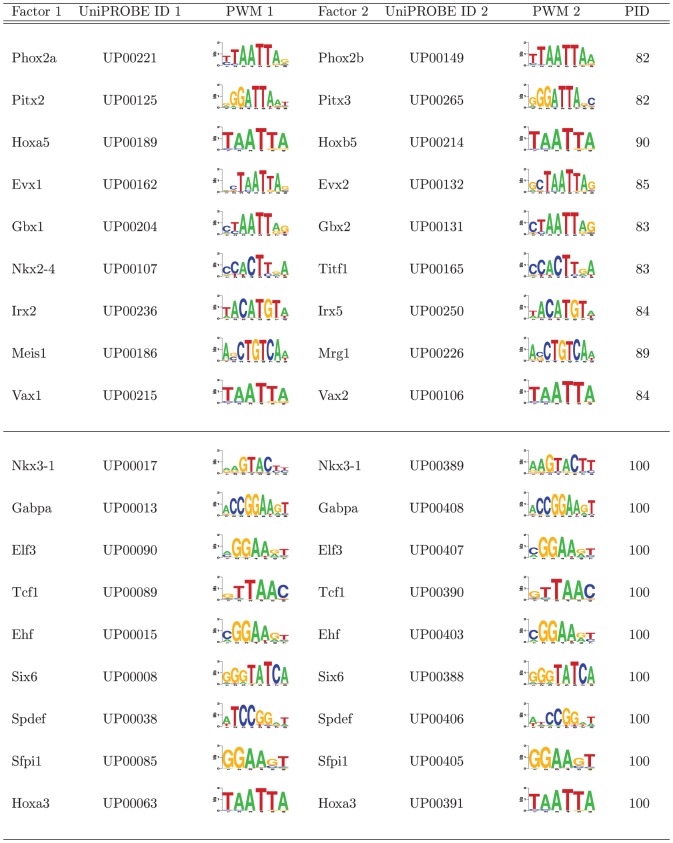
Data set used for validation of domain-level TF-DNA specificities. The top portion contains gene names, UniPROBE identifiers, and truncated position weight matrices for domain-identical transcription factor pairs (test set). The bottom portion contains completely-identical transcription factor pairs with replicate PBM data (control set). PID is the percent identity between the insert sequences of the transcription factor pairs used in the PBM experiments. Sequence logos were created using WebLogo [21].

Protein binding microarray (PBM) data were used to evaluate the proposed domain-level approach as they provide uniform and unbiased measures of sequence specificities for all DNA 10 mers [19]. A publicly available PBM dataset from UniPROBE [20], summarized in Figure 1, was compiled to validate that position weight matrix (PWM) scoring of domain-identical transcription factor pairs (test set) exhibit comparable DNA binding specificity prediction to PWM scoring of completely-identical transcription factor pairs with replicate PBM data (control set). While PBM data alone could be used to validate the present domain-level approach, we were specifically interested in demonstrating the transferability of PWM models derived from domain-identical transcription factors and have therefore assessed PWM scoring with respect to experimental PBM data.

Position weight matrices, generated by the Seed-and-Wobble algorithm [19] and available in UniPROBE, were used for scoring PBM DNA sequences. Due to the short length of PBM DNA sequences, and to minimize edge effects in scanning, a threshold of 0.75 bits as defined in [21] was applied to the beginning and end of all weight matrices to remove positions with low information content. Some positions with information content less than 0.75 bits were retained to maintain equal weight matrix length between transcription factor pairs. The resulting PWMs, shown in Figure 1, were then used to calculate the maximum score from the unique forward and reverse complement sequences of each PBM probe according to the method of Thijs et al. [22].

The PWM:PBM pairs used in validation of the present domain-level approach were established as follows. *Self scoring* of completely-identical pairs (*e.g.*, PWM model derived from UP00017 scored and evaluated against the PBM data of UP00017) and domain-identical pairs (*e.g.*, PWM model derived from UP00221 scored and evaluated against the PBM data of UP00221) was performed to establish an upper limit on the ability for computational scoring with a PWM model to recapitulate the fluoresence intensities of the PBM experiment. *Cross scoring* of completely-identical pairs (*e.g.*, PWM model derived from UP00017 scored and evaluated against the PBM data of UP00389) and domain-identical pairs (*e.g.*, PWM model derived from UP00221 scored and evaluated against the PBM data of UP00149) then allowed for a relative comparison to the self scoring metrics for both the completely-identical transcription factor pair sets and the domain-identical transcription factor pair sets. The present domain-level approach was thereby considered validated if the performance of self and cross scores are comparable regardless of whether the transcription factor pair was completely-identical or domain-identical.

For each given PWM:PBM pairing, the Spearman correlation coefficients were calculated between the maximum PWM score for each PBM probe sequence and the corresponding experimental PBM probe fluorescence intensity. Precision analysis was also conducted to ensure that the Spearman correlation coefficient performance between domain-identical and completely-identical transcription factor pairs was not attributed to distinct clusters of bound and unbound probes in the PBM data. Similar to Chen et al. [23], precision was calculated as the number of top *n* PWM-based scores and PBM probe intensities in common, where *n* varied from 1 to the number of PBM probes.

## Results and Discussion

### Quantifying increased coverage

**Figure 2:**
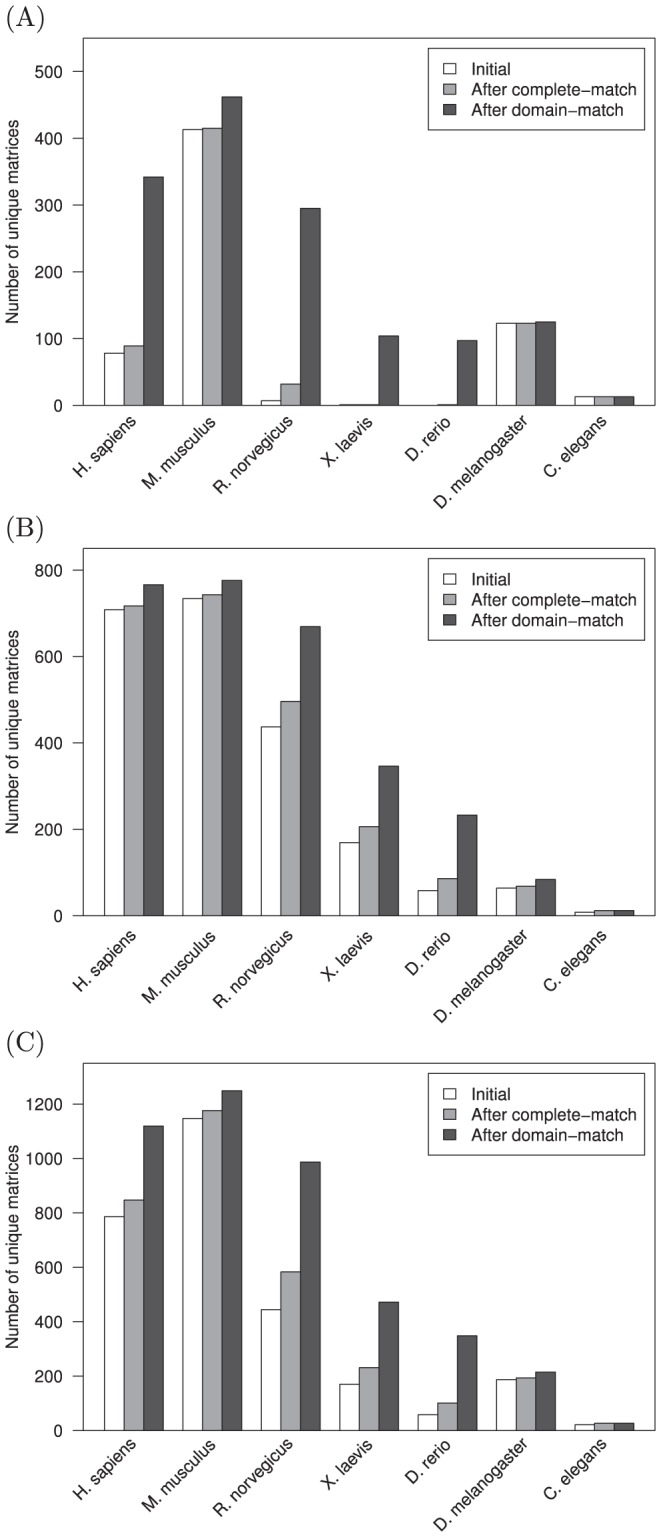
The number of position weight matrices for select organisms before and after homology mapping. The number of matrices that are initially associated with each organism is compared to the number following mapping of transcription factors with completely-identical sequences, as well as the increase following identical DNA binding domain-level mapping for the (A) JASPAR, (B) TRANSFAC, and (C) JASPAR & TRANSFAC databases. The JASPAR and TRANSFAC databases initially contained PWMs from 124 different species, compared to 1578 species following domain-level homology mapping. In particular, significantly increased PWM coverage is possible through domain-level mappings for the open-access JASPAR database.

Owing to the species-centric view that is frequently taken in studying regulatory networks, we assessed the increase in unique position weight matrices (PWMs) associated with each given species enabled by the present domain-level homology mapping approach with respect to existing curation in the JASPAR and TRANSFAC databases. This domain-level homology mapping was also compared to the mappings for complete TF sequence matches. As seen in Figure 2, the magnitude of increased coverage is highly variable from one species to another, though generally higher for the domain-level homology search. Of particular note is the increase in PWM coverage for *H. sapiens*, *R. norvegicus*, and many others in the open-access JASPAR database. This gain demonstrates the potential to provide more thorough species-associated PWM coverage, and consequently regulatory network investigation, using readily available and existing resources. While Figure 2 highlights the increase in PWM coverage for several commonly studied species, this is far from inclusive of the breadth of species with available PWMs. The JASPAR and TRANSFAC databases combined contain PWMs from 124 different species. With the present domain-level homology mapping approach, the number of represented species is increased over an order of magnitude to 1578.

**Figure 3:**
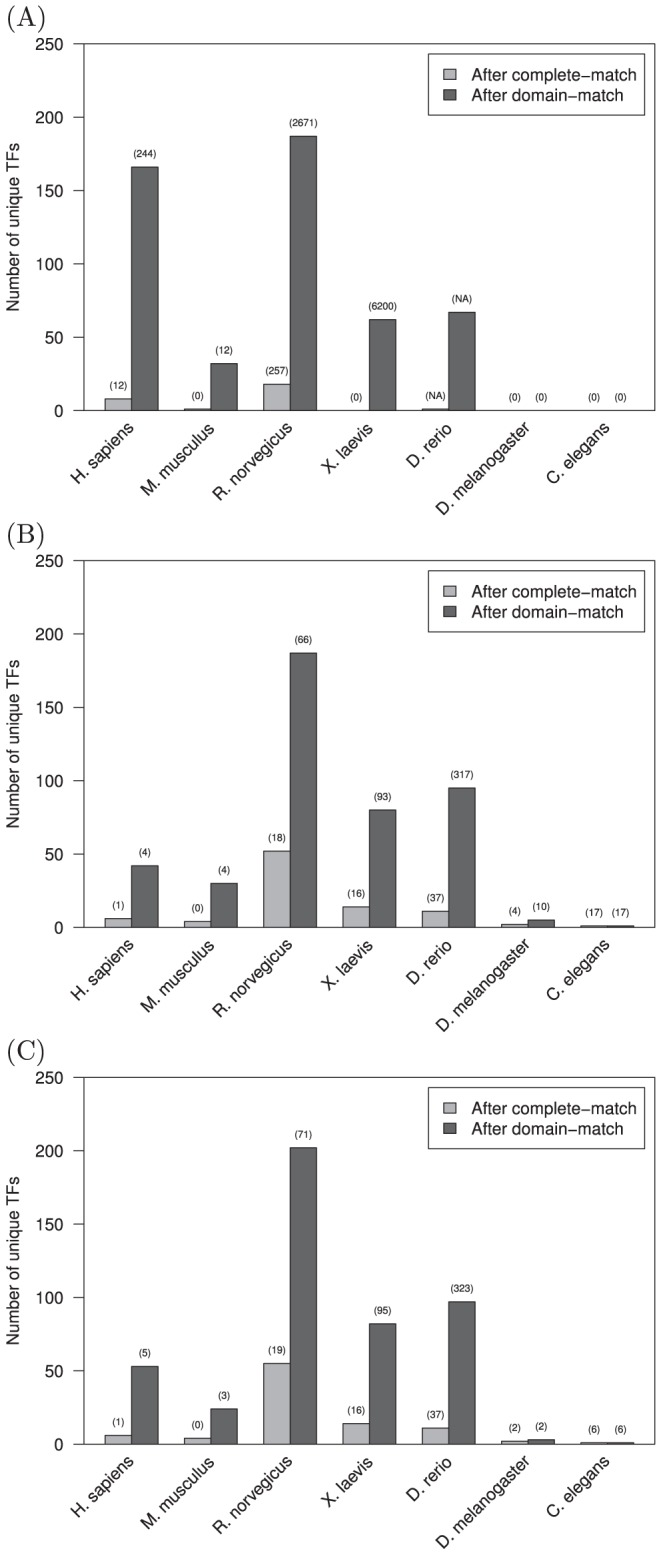
The number of unique transcription factors with position weight matrices (PWMs) resulting from domain-level homology mappings that did not previously have any associated PWMs. The number of unique factors resulting from mapping between completely-identical sequences is compared to the number of factors resulting from identical DNA binding domain-level mapping for the (A) JASPAR, (B) TRANSFAC, and (C) JASPAR & TRANSFAC databases. The number in parenthesis above each bar is the percentage increase above the initial annotated total number of unique transcription factors with PWMs. Significantly increased species-associated transcription factor coverage is enabled by domain-level mappings rather than the typical restriction to complete sequence matches.

In addition to an overall increase in PWM coverage, we determined the number of unique transcription factors with PWMs resulting from domain-level homology mappings that did not previously have any associated PWMs (Figure 3). Significantly increased species-associated transcription factor coverage is enabled by domain-level mappings rather than the typical restriction to complete sequence matches. These unique transcription factors, representative of cross-species DNA binding domain homology matches, enable increased investigation of TF-DNA interactions and regulatory networks. This further demonstrates the utility in applying domain-level homology mappings to readily available information from existing and open-access resources such as JASPAR.

### Method validation

The Spearman correlation coefficients as well as precision curves were calculated between PWM-based scores and experimental PBM fluorescence intensities for all domain-identical and completely-identical transcription factor pairs in Figure 1.

**Figure 4:**
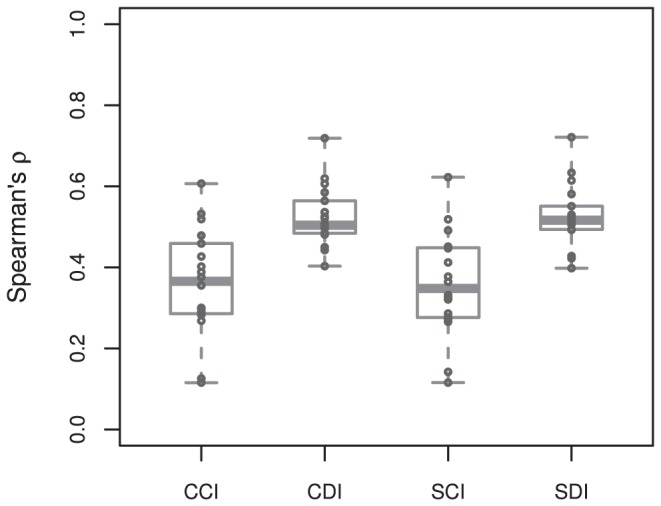
Spearman correlation coefficients ( 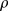
 **) for position weight matrix (PWM) scanning of transcription factor pairs and their accompanying experimental protein binding microarray (PBM) fluorescence intensities.** Transcription factor pair groupings, as in Figure 1, were cross scans of completely-identical pairs (CCI), cross scans of domain-identical pairs (CDI), self scans of completely-identical pairs (SCI), and self scans of domain-identical pairs (SDI). Each point represents a PWM:PBM pairing as described in the Methods. The transcription factor Elf3 (UniPROBE identifiers UP00090 and UP00407) was an outlier with the lowest correlation coefficients. The lower correlation coefficients for these identifiers is likely due to the transcription factor Elf3 having two different DNA binding domains.

**Correlations:** Spearman correlation coefficients (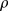) between PWM scores and experimental PBM fluorescence intensities (Figure 4) were calculated for cross scoring of completely-identical pairs (CCI), cross scoring of domain-identical pairs (CDI), self scoring of completely-identical pairs (SCI), and self scoring of domain-identical pairs (SDI) listed in Figure 1. In comparing the completely-identical and domain-identical correlation coefficients, UniPROBE identifiers UP00090 and UP00407 were outliers with the lowest correlation coefficients. Since PWMs are generally a composite of the entire transcription factor and not individual domains, the lower correlation coefficients for these identifiers in both self and cross PWM scoring is likely due to the transcription factor Elf3 having two DNA binding domains (AT hook and Ets), whereas all other transcription factors in the present data set contained a single DNA binding domain. With the exception of these outliers, the comparable performance between completely-identical and domain-identical pairs provided preliminary validation of the present domain-level approach. Additionally, the distributions of (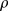) for self and cross scores are comparable regardless of whether the transcription factor pair is completely-identical or domain-identical (*e.g.*, CCI and SCI distributions are equivalent, and CDI and SDI distributions are equivalent).

**Figure 5:**
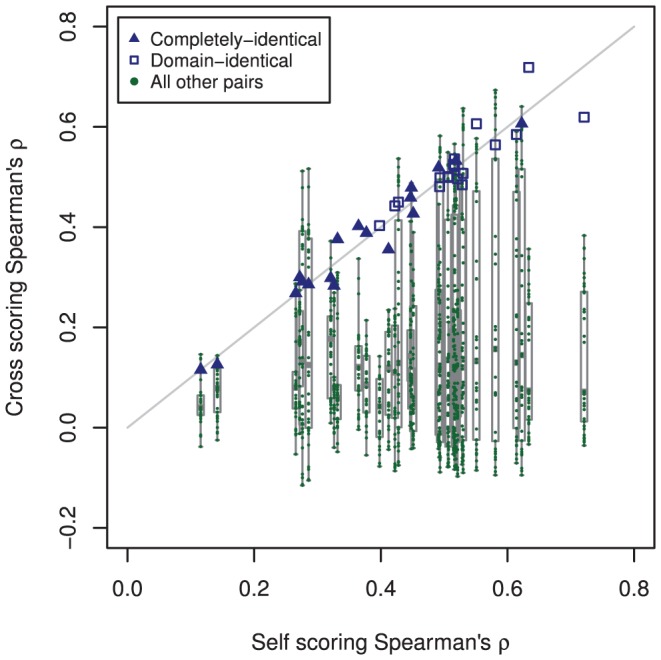
Self and cross Spearman correlation coefficients ( 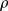
 **) between position weight matrix-based scores and experimental PBM fluorescence intensities.** The blue points are the completely-identical and domain-identical transcription factor pairs of Figure 1. The alignment of blue points along the gray diagonal line demonstrates the comparable performance of PWMs derived from completely-identical and domain-identical transcription factor pairs, whereas the magnitude of 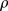 is an indication of how well the PWM captures the DNA binding properties of the transcription factor. As a point of comparison, the correlation coefficients for all other pairwise sets of transcription factors were calculated. The green points below the gray diagonal are indicative of PWMs from other transcription factors that failed to capture the DNA binding properties in the PBM data. Green points near the diagonal resulted from other transcription factors within the same domain family (*e.g.*, homeodomain) that have similar PWMs and, therefore, DNA binding properties. UniPROBE identifiers UP00017 and UP00389 were significantly outperformed by other PWMs in the data set (see text for details).

While Figure 4 provided a summary view of correlation coefficients for self and cross scoring of domain-identical and completely-identical transcription factor pairs, we were interested in further evaluating the pairwise self and cross scoring performance of PWM scoring compared to experimental PBM data (Figure 5).

The magnitude of 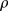 for a given self scoring was an indication of how well the PWM captures the DNA binding properties of the transcription factor. Comparable values for 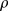 between cross and self scoring demonstrated the ability of PWMs derived from independent PBM data to equivalently capture these DNA binding properties. This is represented by the alignment of completely-identical and domain-identical transcription factor pairs (blue points) along the gray diagonal line. The green points below the gray diagonal are indicative of PWMs from other transcription factors that, as anticipated, failed to capture the DNA binding properties in the PBM data. Green points near the diagonal resulted from other transcription factors within the same domain family (*e.g.*, homeodomain, ETS, *etc.*) that have similar PWMs and, therefore, DNA binding properties.

Interestingly, the Nkx3-1 transcription factor pair (UniPROBE identifiers UP00017 and UP00389) was significantly outperformed by PWMs derived from other transcription factors in the data set. This resulted from the existence of two possible motifs for UP00017 and one motif for UP00389. When the matching UP00017 and UP00389 motif (AAGTACTT) was used in scoring, correlation coefficients near 0.27 were achieved for both factors. When the alternative UP00017 motif was used (TTAAGTGG), correlation coefficients above 0.5 were achieved for both Nkx3-1 identifiers. The improved performance achieved by the TTAAGTGG motif was comparable to the highest correlation coefficients achieved by other PWMs scored against the Nkx3-1 PBM data. These other high performing PWMs were from other homeodomain transcription factors in the data set that possess motifs similar to the alternative Nkx3-1 motif. This is in agreement with the work of Alleyne et al. [24], who demonstrated that the full DNA binding specificity of uncharacterized TFs can be predicted on the basis of similarity in protein sequence alone, given the sequence specificity of closely related members of the same transcription factor family and knowledge of the DNA-contacting residues.

**Figure 6:**
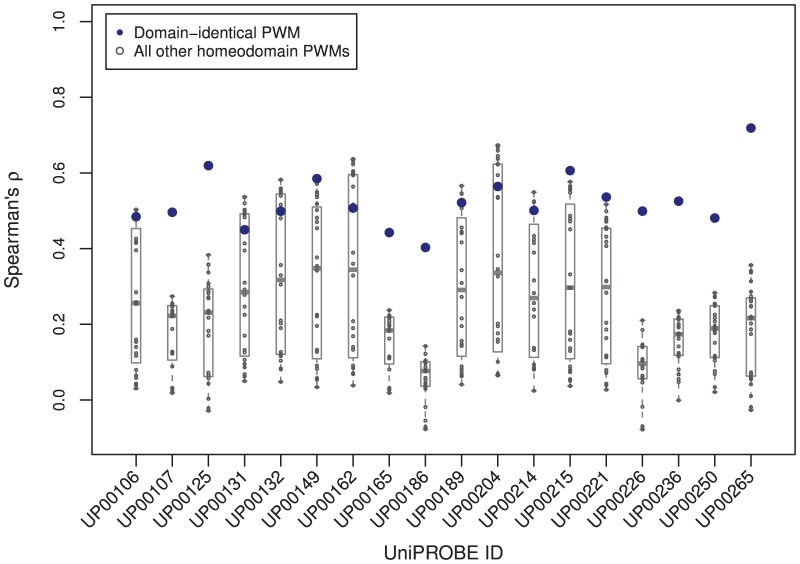
The distribution of Spearman correlation coefficients for the domain-identical PWM and all other PWMs from the same homeodomain family for each TF from the test set in **Figure 1**. In each case, the correlation coefficient for the domain-identical PWM either clearly outperforms or is in the cluster of top performing PWMs, demonstrating that domain-identical PWMs capture the DNA sequence affinity and specificity of transcription factors better than considering the TF family alone.

We also assessed the ability for domain-identical PWMs to predict TF-DNA binding properties more accurately than PWMs from non-identical transcription factors within the same family. Each TF from the test set (top section of Figure 1), all of which are homeodomains, were scored with the PWM from the domain-identical match, and the resulting Spearman correlation coefficients were compared to the results of all other homeodomain PWMs in the data set. The distribution of correlation coefficients for the domain-identical PWM and all other homeodomain PWMs for each TF from the test set is shown in Figure 6. In each case, the correlation coefficient for the domain-identical PWM either clearly outperforms or is in the cluster of top performing PWMs. This provides further support to the notion that, due to select residues within the DNA binding domain(s) of the TF interacting directly with DNA, domain-identical PWMs capture the DNA sequence affinity and specificity of transcription factors better than expected by considering the TF family alone.

**Figure 7:**
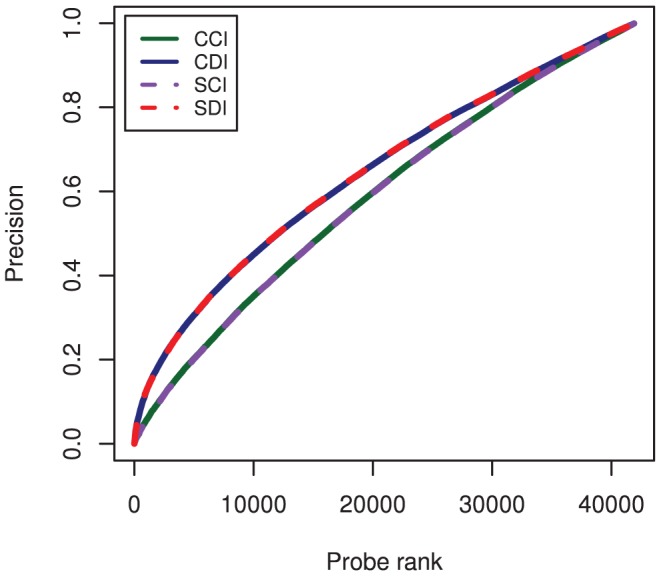
Average precision curves, calculated as the number of top *n* position weight matrix-based scores and experimental PBM fluorescence intensities in common. Precision curves were generate for cross scoring of completely-identical pairs (CCI), cross scoring of domain-identical pairs (CDI), self scoring of completely-identical pairs (SCI), and self scoring of domain-identical pairs (SDI) listed in Figure 1. The average precision is nearly exactly overlaying for CCI and SCI, as well as CDI and SDI, owing to the ability of self and cross PWM scans to equivalently capture the DNA binding properties in the PBM data. As with the Spearman correlation coefficients in Figure 4, the average precision for the domain-identical data set actually outperformed the completely-identical transcription factor pair scoring, reflecting the more challenging nature of the completely-identical data set (see text for details).

**Precision:** To ensure that the comparable Spearman correlation coefficient performance between domain-identical and completely-identical pairs was not attributed to distinct clusters of bound and unbound probes in the PBM data, we also evaluated the precision curves as the number of top *n* PWM-based scores and PBM probe intensities in common. The average precision between position weight matrix-based scores and experimental PBM fluorescence intensities (Figure 7) was calculated for cross scoring of completely-identical pairs (CCI), cross scoring of domain-identical pairs (CDI), self scoring of completely-identical pairs (SCI), and self scoring of domain-identical pairs (SDI) listed in Figure 1. The average precision is nearly exactly overlaying for CCI and SCI, as well as CDI and SDI, owing to the ability of self and cross PWM scoring to equivalently capture the DNA binding properties in the PBM data. As with the Spearman correlation coefficients in Figure 4, the average precision for the domain-identical data set actually outperformed the completely-identical transcription factor pair scoring. This is largely a reflection of more challenging cases in the completely-identical data set, which included one transcription factor pair with multiple DNA binding domains (Elf3) and another transcription factor pair with two possible PWM motifs (Nkx3-1). The comparable precision between domain-identical and completely-identical pairs provides further validation of the present domain-level approach and transferability of PWM models for domain-identical transcription factors.

## Conclusions

Based on correlation and precision assessments of position weight matrix (PWM) scores and experimental protein binding microarray (PBM) fluorescence intensities, we have demonstrated that the DNA binding properties of homologous transcription factors with identical DNA binding domains are equivalent. Accordingly, we have developed an automated pipeline for identifying and cross-mapping homologous transcription factors with identical DNA binding domains.

By applying this domain-level homology search to transcription factors with existing PWMs in the JASPAR and TRANSFAC databases, we were able to significantly increase species-associated PWM coverage, assign PWMs to transcription factors that did not previously have any associations, and increase the number of represented species with PWMs over an order of magnitude. These gains demonstrate the potential for more thorough species-associated investigation of protein-DNA interactions using existing resources, particularly with the open-access JASPAR database.

The PWM scoring results highlight the challenging nature of transcription factors that contain multiple DNA binding domains, as well as the impact of motif discovery on the ability to predict DNA binding properties, indicating areas for future development. Additionally, the generally low correlation and precision of PWM scoring with respect to experimental PBM fluorescence intensities demonstrates the limitation of PWMs as a model for TF-DNA binding affinities highlighting the opportunity for alternative approaches to utilizing PBM data [25].

While the present work focuses on transcription factors with available PWMs, additional data types can be easily integrated into the domain-level homology search pipeline. Similarly, the method is suitable for identifying domain-level homology mappings to enable genome-scale analyses and comparative genomics of transcription factor-DNA interactions.

### Availability

The DNA binding domain-level homology search method and resulting UniProt-PWM mappings for JASPAR and TRANSFAC are publicly available at http://dodoma.systemsbiology.netdodoma.systemsbiology.net. Additionally, a web API has been developed, including sample scripts for programmatic batch submission and analysis pipelines.
